# Functional Phylogenetics Reveals Contributions of Pleiotropic Peptide Action to Ligand-Receptor Coevolution

**DOI:** 10.1038/srep06800

**Published:** 2014-10-28

**Authors:** Hongbo Jiang, Zhaojun Wei, Ronald J. Nachman, Michael E. Adams, Yoonseong Park

**Affiliations:** 1 Key Laboratory of Entomology and Pest Control Engineering, College of Plant Protection, Southwest University, Chongqing 400715, P. R. China; 2 Department of Entomology, Kansas State University, Manhattan, Kansas 66506, United States; 3 Insect Control and Cotton Disease Research Unit, Southern Plains Agricultural Research Center, USDA, 2881 F/B Road, College Station, TX 77845, USA; 4 Departments of Entomology and Cell Biology & Neuroscience, 2103 Biological Sciences Building, University of California, Riverside, CA 92521, USA

## Abstract

The evolution of peptidergic signaling has been accompanied by a significant degree of ligand-receptor coevolution. Closely related clusters of peptide signaling molecules are observed to activate related groups of receptors, implying that genes encoding these ligands may orchestrate an array of functions, a phenomenon known as pleiotropy. Here we examine whether pleiotropic actions of peptide genes might influence ligand-receptor coevolution. Four test groups of neuropeptides characterized by conserved C-terminal amino acid sequence motifs and their cognate receptors were examined in the red flour beetle (*Tribolium castaneum*): 1) cardioacceleratory peptide 2b (CAPA); CAPAr, 2) pyrokinin/diapause hormone (PK1/DH); PKr-A, -B, 3) pyrokinin/pheromone biosynthesis activating hormone (PK2/PBAN); PKr-C, and 4) ecdysis triggering hormone (ETH); ETHr-b. Ligand-receptor specificities were established through heterologous expression of receptors in cell-based assays for 9 endogenous ligands. Based on ligand-receptor specificity analysis, we found positive pleiotropism exhibited by ETH on ETHR-b and CAPAr, whereas PK1/DH and CAPA are more highly selective for their respective authentic receptors than would be predicted by phylogenetic analysis. Disparities between evolutionary trees deduced from receptor sequences vs. functional ligand-receptor specificities lead to the conclusion that pleiotropy exhibited by peptide genes influences ligand-receptor coevolution.

Diffusible peptide signaling molecules acting via cell-surface receptors are evolutionary innovations for functional coordination of organ systems in animals. Partnerships between ligand and receptor persist through a process of ligand-receptor coevolution^1^. Among the diversity of peptides are closely related ligand groups that mediate distinct physiological functions.

Genome analysis reveals that acquisition and/or specialization of phenotypic traits accompanying speciation often includes appearance of new neuropeptide signaling pathways (i.e., tachykinin and natalisin signals^2^). Stability of a newly evolved ligand-receptor signaling pathway may require its rapid sub- or neo-functionalization^3^,^4^ for minimization of functional redundancy, for example through changes in spatial or temporal expression patterns. However, such a role for ligand-receptor coevolution in diversifying evolution is only one possible outcome. For example, actions of the neuropeptide FMRFamide on multiple receptors, including the receptor for the related neuropeptide FLRFamide, known as myosuppressin, in the silkworm *Bombyx mori* hints at likely evolutionary advantages associated with “cross-talk” or pleiotropism inherent in signaling strategies^5^. Pleiotropism occurs when a single gene influences multiple phenotypic traits. In the context of peptide signaling, a single peptide gene product signaling via distinct receptors may lead to multiple physiological and hence phenotypic outcomes. Such pleiotropic peptide action therefore could be an important evolutionary force. It may be time to recognize that evolution selects not just single ligand-receptor partners, but webs of interdigitating peptide signaling networks that orchestrate physiological states involving multiple organ systems and diverse functions.

Here we describe possible roles of pleiotropism in evolution of a neuropeptide cluster characterized by the C-terminal amino acid sequence motif “-PRXamide” and corresponding receptors in the red flour beetle, *Tribolium castaneum*. Our data support a role for pleiotropism in evolution of ligand-receptor interactions.

## Results and Discussion

We investigated influences of pleiotropism in ligand-receptor coevolution by comparing evolutionary history according to alternative criteria: 1) amino acid sequence similarity and 2) ligand potency. According to the hypothesis of neutral evolution, evolutionary patterns should be similar whether gauged by molecular divergence of genes or by functional specificities of ligand-receptor interactions.

### The PRXamide Peptide Cluster

The PRXamide cluster of neuropeptides has diversified considerably in insects, while its mammalian counterpart is confined to neuromedin U (F-x-P-R-x-amide) and possibly thyrotropin releasing hormone, based on molecular phylogeny of their corresponding receptors^1^. Hereafter, consensus sequences are represented according to Prosite syntax rules, where amino acids are separated by a hyphen (-), x denotes wild card amino acids, and a square bracket denotes a small number of possible amino acid assignments at a given position.

**Figure 1:**
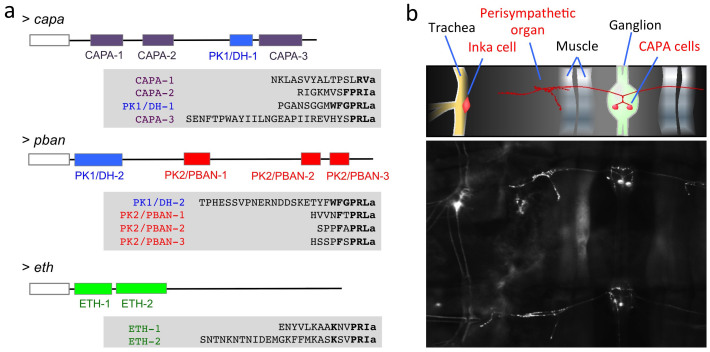
Three genes encoding –PRXamide peptide in the *T. castaneum* (a) and abdominal segments of a larva showing endocrine organs for ETH and CAPA visualized by immunohistochemistry (b). (a) Schematic diagrams for CAPA, PBAN, and ETH genes are shown on the top line of each gene product. Mature peptides are shown as color coded boxes for each peptide family. Empty boxes to the left in each diagram indicate signal peptides. Sequences for each putative mature peptide are aligned according to conserved C-terminal amino acid sequence motifs marked in bold. The italic used in the N-terminus of sequence ETH-2 indicates uncertainty with the canonical cleavage signal in the middle of the ETH-2 as GK. This study used the shorter version of ETH-2 starting with FFM in the alternative predictions. (b) Immunostaining of –PRXamide-expressing neurons. The top inset is a schematic diagram depicting stained elements visible below. Ventral larval abdominal segments are shown for the CNS and trachea containing the cells for CAPA and for ETH (Inka cell), respectively, and for the perisympathetic organ, which serves as a neurohemal release site for the neuropeptide CAPA.

The insect PRXamide cluster includes four families of neuropeptides (Fig. 1a): cardioacceleratory peptide 2b (hereafter named as CAPA), pyrokinin/diapausing hormone (PK1/DH or PK1), pyrokinin/pheromone biosynthesis activating hormone (PK2/PBAN or PK2), and ecdysis triggering hormone (ETH). Functions of this peptide group have been intensively studied over the past several decades, although information remains fragmented in some insect species and is limited to results from a few specific physiological assays, often chosen for ease of use rather than authentic physiological function(s) that are often unknown. It is notable that PRXamide peptides are predominantly hormonal signaling molecules, which allows for interactions with a broad diversity of receptors and for possible evolutionary selection based on adaptive influences of pleiotropism.

The CAPA family, named by virtue of cardioacceleratory activity, includes periviscerokinin, which shows myotropic activities through neurohemal release from segmental perisympathetic organs^6^. This family of peptides is characterized by the general C-terminal consensus sequence [LI]-x(2)-F-P-R-[VI]-amide (where x(2) means any two amino acids), with minor variations^7^. PK1/DH, known for its role in embryonic diapause in *B. mori*^8^,^9^ and for breaking pupal diapause in heliothine moths^10^,^11^,^12^,^13^, has consensus sequence W-F-G-P-R-L-amide with minor variations. PK2/PBAN is the well-known pheromone biosynthesis activating neuropeptide in Lepidoptera^14^,^15^ with consensus sequence F-x-P-R-L-amide. Notably, this sequence lacks the PK1/DH sequence motif. Finally, ETH targets numerous central peptidergic neuronal ensembles for activation of sequential preecdysis and ecdysis behaviors^16^,^17^,^18^. ETH has the C-terminal consensus sequence K-x(2)-P-R-[IVLM]-amide with minor variations. The nomenclature of neuropeptides described in this study follows that proposed by Coast and Schooley^19^ with minor modifications.

An interesting phenomenon in the PRXamide peptide group that possibly relates to functional pleiotropism involves two different genes, *capa* and *pban*, which encode mixed ligand arrays consisting of CAPA, PK1/DH, and PK2/PBAN sequences (Fig. 1a). In most insects, the *capa* gene encodes a propeptide precursor containing CAPA and PK1/DH, while the *pban* gene encodes a precursor containing the related PK1/DH sequence and multiple PK2/PBAN-like peptides^20^. For example, the *pban* precursor gene of *Helicoverpa zea* encodes five mature peptides, including one copy of DH, one copy of PBAN and three PBAN-like peptides^14^. In this case, peptide precursors containing multiple peptides may undergo differential postranslational processing in different cell types, generating a diversity of peptide signals and physiological outcomes.

By way of example, the *T. castaneum capa* gene encodes authentic CAPA (also known as periviscerokinin) with a characteristic C-terminal FPRIa sequence motif (CAPA-2), but also encodes two additional peptides with SLRVamide and SPRLamide C-terminal sequence motifs (CAPA-1 and CAPA-3, respectively), along with one PK1/DH peptide (Fig. 1a). The *pban* gene encodes a PK1/DH peptide together with three peptides belonging to the PK2/PBAN group (PK2/PBAN-1, -2, and -3). The *eth* gene encodes two closely related ETH peptides: ETH-1 and ETH-2 (Fig. 1a). This evolutionary radiation of PRXamide peptides raises the possibility of pleiotropic actions on multiple receptors.

We also investigated proximity of PRXamide peptide release sites through immuno staining using an antiserum that cross-reacts with all members of the group. This suggests that the perisympathetic organ and Inka cell function as CAPA and ETH release sites, respectively in larval *T. castaneum* (Fig. 1b). The fact that these two release sites are in close proximity raises the possibility that potential functional interactions exist between these two closely related endocrine signals.

### Cognate GPCRs of the PRXamide Peptide Cluster

The general consensus among receptor annotations for PRXamide neuropeptides across a wide range of insect orders places CAPA, PK, and ETH receptors into distinct categories based on sequence homologies consistent with receptor specificities for their respective ligands. In a similar manner, PK receptors can be sub-divided into distinctive PK1/DH and PK2/PBAN receptors, but with larger degrees of ligand cross-activities^1^,^21^,^22^,^23^,^24^,^25^,^26^,^27^,^28^.

**Figure 2:**
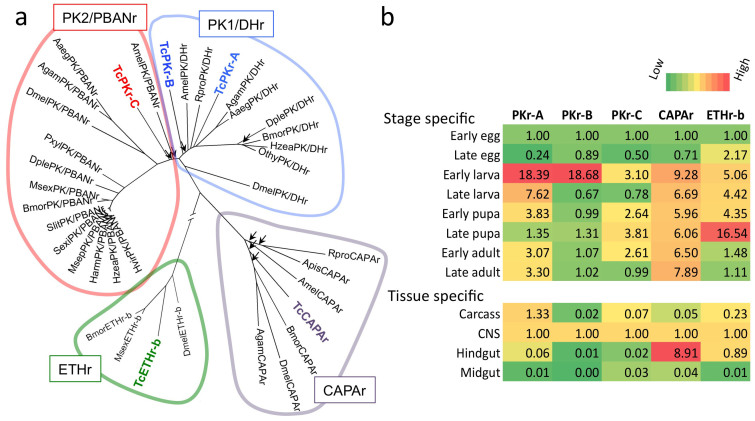
Five *T. castaneum* GPCRs belonging to the cluster of receptors for –PRXamide peptides (a) and their expression patterns (b). (a) A total of five receptors, TcPKr-A, -B, and –C, TcCAPAr, and TcETHr appear within a group of four by phylogenetic criteria: PK2/PBANr, PK1/DHr, CAPAr, and ETHr. Arrowheads indicate bootstrapping values lower than 75, while all other nodes are supported by bootstrapping values higher than 75. Note that ETHr cluster is considerably separated from other clades. (b) Levels of mRNA for each receptor in different stages and tissues. Tissue specific expression data is for the late larval stage. The data show relative values for early egg in the stage specific data and for the CNS in the tissue specific data after normalization by rps3 expression.

Information available from the genomic sequence of *T. castaneum* supports classification of five G protein-coupled receptors (GPCRs) in the PRXamide group^29^,^30^ (Fig. 2a): one CAPA receptor (CAPAr), three PK receptors denoted here as PKr–A, -B, and -C, and one ETH receptor that has alternatively spliced forms ETHr–a, and ETHr–b. Based on our sequencing results, TcPKr-C and TcCAPAr match sequences predicted from the genome database. However, TcPKr-A has an eleven amino acid insertion and TcPKr-B has a three amino acid extension at the 5′ end not found in genome predictions. We updated the sequence information in GenBank on the NCBI website, and corresponding accession numbers are provided in Table 1. Phylogenetic analyses clearly distinguish CAPAr, PKr, and ETHr (Fig. 2a). Within the PKr group, TcPKr-A and TcPKr-B belong to the PK1/DH receptor cluster, whereas TcPKr-C forms an unstable branch on the basal lineage of the PK2/PBAN cluster with low bootstrapping supports (Fig. 2a).

Our results on stage- and tissue-specific expression patterns show that TcPKr-A and B, which are the most similar to each other in sequence, also show similar stage-specific expression patterns (Fig. 2b). TcPKr-B and –C exhibit similarly high expression in the central nervous system (CNS). TcPKr-A,-B,-C and TcCAPAr are all highly expressed in early larval stages. It is noteworthy that TcCAPAr is highly expressed in the hindgut, consistent with the hormonal diuretic function of CAPA previously documented in a number of insect species^6^,^31^,^32^. However, these expression data apparently are too limited in resolution to make inferences regarding evolutionary significance.

### Ligand Specificities of PRXamide GPCRs

**Figure 3:**
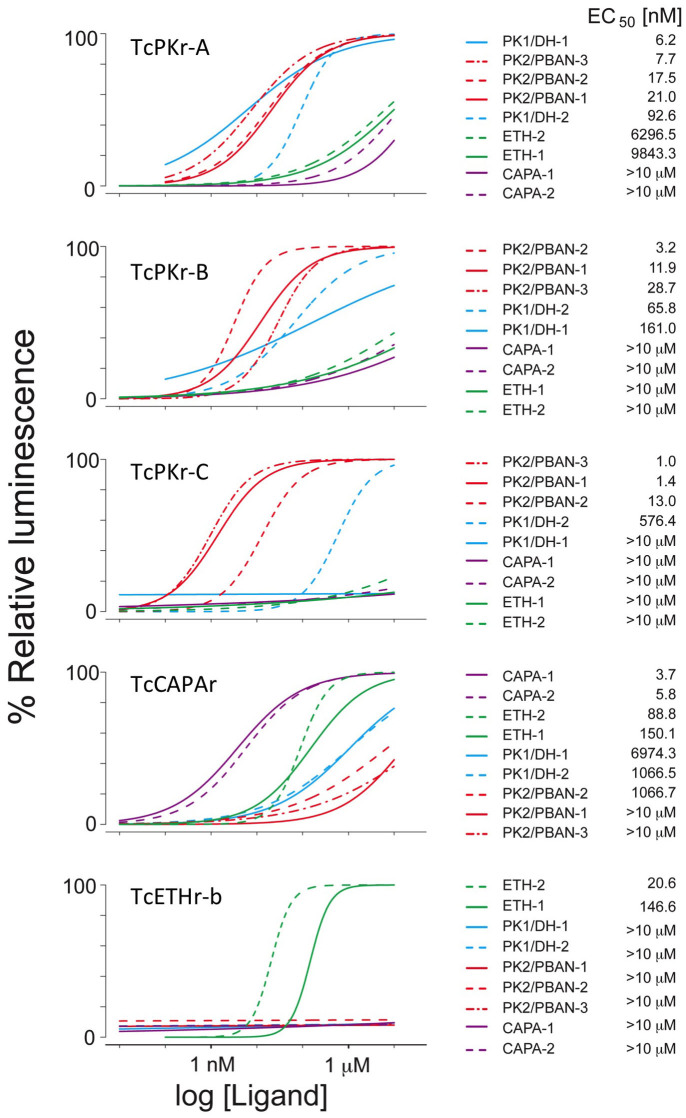
Dose-response curves of five different PRXamide receptors of *T. castaneum* for nine endogenous ligands. Left panel shows dose-response curves for each receptor with 9 putative endogenous ligands. Right panel shows names of ligands and their EC_50_s in rank order. Values of data points are found in Supplementary Data 1.

We characterized all PRXamide GPCRs through assays of 9 endogenous ligands. Ligand activities were quantified by detection of luminescence responses from CHO cells transiently transfected with GPCR (see Methods and Materials section for details). Dose-response curves were generated by logistic fitting of the log transformed ligand concentrations ranging from 0.01 nM to 10 μM (Fig. 3). All results, shown as averages, were replicated in three independent experiments (see Supplementary Data 1 for details). TcETHr-b, a splicing variant used in this study, showed relatively lower activity than those of other receptors, likely due to inefficient coupling to the reporter system.

All GPCRs showed similar levels of sensitivity to their respective authentic ligands (low nanomolar EC_50_ values; Fig. 3), except for ETHr-b (EC_50_ values were 146.6 and 20.6 nM for TcETH1 and TcETH2, respectively; Fig. 3). Among three different *Tribolium* PK receptors, TcPKr-C was activated by only TcPK2/PBANs and activated by high concentrations of TcPK1/DH-2, but not by TcPK1/DH-1. Therefore, our results suggest that TcPKr-A and –B are activated by both TcPK/DH and TcPK/PBANs, while TcPKr-C is likely the authentic receptor specific for TcPK2/PBANs. TcCAPA and TcETH ligands had little or no activity on PK receptors. For TcCAPAr, the two TcCAPA ligands were highly potent with significant levels of cross activities of TcETHs. TcETHr-b was highly specific for TcETH ligands.

### Pleiotropism

**Figure 4:**
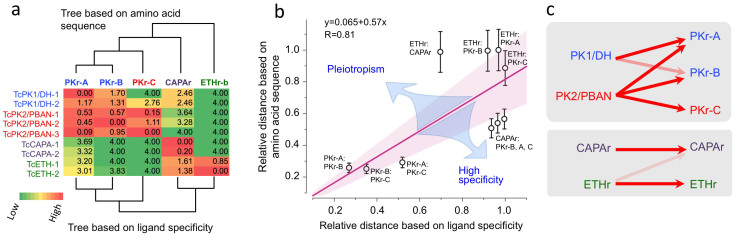
Relative activities of endogenous ligands on five different PRXamide receptors (a), a plot showing relatedness of the sequence diversities and ligand specificities of PRXamide receptors (b), and a simplified diagram showing the asymmetric cross activities of ligands on target receptors (c). (a) Values in the matrix are for log (EC_50_) normalized by the lowest EC_50_ for each receptor. The evolutionary tree based on GPCR amino acid sequence (top) is different from the tree inferred based on the ligand specificity (bottom). Color codes for each cell are low activity (green) to high activity (red). See Materials and Methods for further details. (b) Pairwise distances between receptors. Comparative distances between the distances based on ligand specificity (X-axis) and based on sequence similarity (Y-axis) for each pair of receptors are plotted. Error bars are for the standard error generated in 1000 bootstrapping in the sequence distances. The distance was normalized by the largest distance as 1. Regressed line is with 95% confidence interval (shaded area). Blue arrow implies directions of evolutionary forces for positive or negative pleiotropism. (c) Values in Figure 4a are used to depict a simplified view of asymmetry in pleiotropism. The line color indicates degree of activity; darker lines depict stronger activity.

Evolutionary distances between PRXamide GPCRs were computed according to two criteria: 1) amino acid sequence similarity and 2) ligand specificity (Fig. 4a and b). Relative ligand activities were normalized to the strongest response of authentic partners as described in the Materials and Methods section (Fig. 4a). Overall, evolutionary distances computed by these two criteria (see Materials and Methods section for details) are well correlated, as the regression line shown in Fig. 4b indicates; this is consistent with the hypothesis of neutral evolution in determination of ligand-receptor evolutionary distances. However, we found a major deviation in the co-relationship of TcCAPAr and TcETHr. Although molecular evolution criteria predict considerable divergence between this receptor pair, functional ligand-receptor specificities predict a much smaller evolutionary distance (Fig. 4b). As a consequence, the evolutionary tree generated with ligand specificity as the determining criterion shows clustering of TcCAPAr and TcETHr (Fig. 4a), in contrast to the tree clustering TcCAPAr and TcPKrs according to amino acid sequence similarity.

The dual action of TcETH on TcETHr and TcCAPAr indicates positive pleiotropism and functional coupling of TcETHr and TcCAPAr (Fig. 4a). TcCAPA is released from segmental perisympathetic organs and likely promotes diuresis and myoactivity *in vivo*^33^, while TcETH triggers a downstream peptide signaling cascade that triggers sequential ecdysis behaviors and associated physiological events including diuresis, myoactivity, and tracheal air-filling^18^. Targets of ETH include kinin neurons that promote diuresis and FMRFamide neurons that regulate myoactivity. Evidence presented here indicates that ETH, in addition to activating downstream peptidergic neurons, also activates related receptors such as CAPAr. Thus actions of ETH precursor gene products on both ETHr and CAPAr suggests that these two signaling pathways arose through concerted evolution through positive pleiotropism.

As shown in the matrices of ligand activities on each receptor (Fig. 4a), cross-activities are not necessarily reciprocal (Fig. 4c). For instance, the activity of TcETH on the TcCAPAr is not complemented by similar potency of TcCAPA on TcETHr. Another noticeable asymmetry occurs between PK1/DHs and PK2/PBANs. Strong activity of PK2/PBANs on all three PKr is asymmetric to the activity of PK1/DHs that has activities only to PKr-A and –B, but not to PKr-C (Fig. 4c). Therefore, CAPA and PK1/DHs act specifically only on their respective authentic receptor, but devoid of activating other receptors. This asymmetry supports the conclusion that divergence of ligand specificity is not random, but a consequence of selective pressure at the functional level for favoring pleiotropism or for high specificity.

We expanded our survey of PRXamide peptide pleiotropism to other species of insects, although only limited data were available to draw comprehensive conclusions. Data from two representatives of the Diptera (*D. melanogaster*^34^ and *An. gambiae*^35^) show that ETH is devoid of activity on CAPAr, in contrast to results of this study. Moderate levels of ETH activity on PKrs are commonly observed in many different insect species, including *D. melanogaster*^21^,^27^, *An. gambiae*^35^, *H. virescens*^26^, and *H. zea*^22^,^23^, in addition to the mild degree of positive pleiotropism we observe in the *T. castaneum*. Discrimination against PK1/DH by the PK2/PBAN receptor occurs in *D. melanogaster*^21^,^27^ and *T. castaneum* (i.e., TcPKr-C in this study), whereas moderate degrees of cross potency of PK1/DH on the PK2/PBAN receptor were observed in *An. gambiae*^35^, *Ae. aegypti*^36^, *B. mori*^24^,^28^, *H. virescens*^26^, and *H. zea*^22^,^23^. When more comprehensive data are available for expanded divergent taxa, evolutionary patterns of pleiotropism may reveal whether such activity is attributable to residual cross-activity (pleiotropism) in the duplicated system or whether pleiotropy was obtained secondarily in the evolutionary process.

Pleiotropic interactions among closely related ligands and corresponding receptors appear to be important in fine tuning orchestrated multi-organ physiological outcomes and consequently may be subject to selection pressures in the evolutionary process. In conclusion, this study provides evidence for influences of pleiotropism in ligand-receptor co-evolution in the PRXamide-receptor cluster. It seems possible that this principle may be expanded and applied to algorithms with greater effectiveness and selectivity in drug design targeting GPCRs.

## Methods

### Insects and chemicals

*T. castaneum* was kept in a 30°C growth chamber under a 16 : 8 (L : D) photoperiod and fed on a diet of wheat flour and Brewer's yeast (10 : 1). Peptides from *T. castaneum* were synthesized by Genescript (Piscataway, NJ). For culture of Chinese Hamster Ovary cells (CHO), DMEM/F12 medium, fetal bovine serum (FBS), Fungizone and Penicillin/Streptomycin, and coelenterazine for aequorin functional assay were purchased from Gibco cell culture at Life Technologies^TM^ (Grand Island, NY). TransIT-LT1 Transfection Reagent (Mirus Bio LLC, Madison, WI) was used for transient transfections.

### Molecular cloning

Total RNA was isolated from the whole body of six individuals of *T. castaneum* last larval instar using TRI reagent (Ambion), treated by DNase I (Ambion) to eliminate genomic DNA, and followed by the Phenol-Chloroform extraction. First strand cDNA was synthesized by SuperScript^TM^II First-Strand Synthesis System for RT-PCR with random hexamer in the total volume 20 μL, according to manufacturer's instructions (Invitrogen Life Technologies). The 1^st^ strand cDNA was used as template to amplify target PRXamide receptors utilizing high fidelity DNA polymerase PrimeSTAR^TM^ HS (Takara). Primers for all five GPCRs were designed based on *T. castaneum* genome data. For CAPA and three pyrokinin receptors (TcPKr-A, -B and -C), we started with four predictions including TC007170, TC011171, TC011320 and TC011318, respectively. Meanwhile, we started with the experimentally confirmed sequence (GenBank accession number is EF222294) for ETHr-b. Functional expression of ETH-a failed for unknown reasons. All primer information is provided in Table 1. Reactions were conducted in a volume of 50 μL and included ~50 ng cDNA, 10 μL 5x PrimeSTAR buffer with Mg^2+^, 0.32 mM for each dNTP, 0.2 μM of each primer. The PCR program included 35 cycles: 98°C for 10 sec, 58°C for 10 sec and 72°C for 90 sec with a final extension of 6 min at 72°C. The PCR product was cloned into a pGEMT Easy vector (Promega) and sequenced.

### Sequence analysis and tree construction

Nucleotide sequences and deduced amino acid sequences of the PRXamide receptors were analyzed using DNAMAN7 (LynnonBioSoft). Searches of similar sequences were done with BlastP in the non-redundant protein sequences (nr) database of the NCBI website (http://www.ncbi.nlm.nih.gov/). Sequence alignments were made by ClustalW2 (http://www.ebi.ac.uk/Tools/msa/clustalw2/). Transmembrane helices were predicted using the TMHMM server (http://www.cbs.dtu.dk/services/TMHMM) and only sequences covering transmembrane segments 1 to 7 and their linkers, excluding N- and C-termini of highly diversified regions, were used for the tree (Fig. 4a). A phylogenetic tree was constructed by MEGA5^37^ by applying the Neighbor-Joining method with a bootstrap test of 1000 replications and complete deletion of gaps produced by the alignment. The distances between receptors obtained in the phylogenetic tree were.normalized by the maximum distance as 1 and used the plot value for “relative distance based on the amino acid sequence” (Fig. 4b).

The ligand activity matrix shown in Fig. 4A depicts log_10_-transformed EC_50_s normalized to the most potent authentic ligand for a given receptor using the formula: log{[EC_50_(test ligand)]/log[EC_50_(lowest)]}, where EC_50_(lowest) is the EC_50_ for endogenous partner ligand. In order to avoid artifacts introduced by extremely high EC_50_ values, we set 4 as the maximum value (e.g., EC_50_ of 10^4^ nM) for log transformed EC_50_s. The ligand specificity tree was constructed in Cluster 3.0^19^. The distances based on ligand specificity were obtained by using the R Stats Package Euclidean method in Distance Matrix Computation (http://stat.ethz.ch/R-manual/R-patched/library/stats/html/00Index.html). Relative distances were obtained by normalization to the maximum distance as 1.

### Heterologous expression and functional assay

cDNAs for full GPCR ORFs were inserted into the expression vector pcDNA3.1(+) (Invitrogen) or pcDNA5/FRT (Invitrogen). Methods for expression in Chinese Hamster Ovary (CHO) cell supplemented with aequorin and G-α16 and the assays were performed as previously described^38^. Briefly, cells were transfected using the TransIT-LT1 transfection reagent (Mirus Bio LLC). Thirty hours after the transfection, the cells were collected and preincubated with the coelenterazine (Invitrogen) prior to the functional assay. A serial dilution (10 folds) of the 9 endogenous ligands were applied to the cells. The luminescence caused by the intracellular calcium mobilization were measured in continuous 20 seconds in every half-second interval. The authentic ligands showing most potent activity on the receptors were selected as model ligand for normalization of the luminescence. Based on which, a dose response curve was generated for each receptor by logistic fitting in Origin 8.6 (OriginLab).

### Quantitative reverse transcriptase PCR (Q-PCR)

Expression patterns of GPCRs were examined by Q-PCR. Total RNA was collected from insects at different developmental stages and prepared as described previously: early egg (<24 h), late egg (>24 h), early larva (<24 h post-hatching), late larva (older than 5th instar including prepupa), early pupa (<24 h post-pupation), late pupa (>72 h post-pupation), early adult (<24 h post-eclosion), and late adult (one week old)^39^. To quantify tissue-specific expression, we collected total RNA from each of the following dissected tissues: CNS (central nervous system including brain and ganglia), midgut, hindgut, and carcass excluding the aforementioned tissues. A pool of ten last-instar larvae was used for preparation of each tissue. Total RNA was treated by DNaseI (Ambion). Primers used in Q-PCR were listed in Table 1. 1st strand cDNA for qPCR was synthesized using ImProm-II™ Reverse Transcription System (Promega). Real-time RT-PCR was performed by using the iTaq^TM^ Universal SYBR Green supermix (Biorad) on a CFX Connect real time detection system (Biorad). Data expressed here as relative mRNA levels were normalized to a reference gene *ribosomal protein S3* (*rpS3*, GenBank accession number is CB335975), using the ΔΔCT method^40^. For samples collected from each developmental stage or tissue, we performed three biological replicates. For data analysis, relative expression of the target transcripts in early eggs (EE) served as the calibrator for developmental expression profiling, while target transcript expression in the CNS was employed as the calibrator for tissue specific expression profiling.

### Immunohistochemistry

The antibody (anti-DrmETH1) used for recognition of–PRXamide motifs was raised in a rabbit. The central nervous system from last instar larvae was dissected in ice-cold phosphate-buffered saline (PBS: 137 mM NaCl, 1.45 mM NaH_2_PO4, 20.5 mM Na_2_HPO4, pH 7.2), freed of remaining fat, and fixed in 4% paraformaldehyde at 4°C overnight. Fixed samples were washed in PBS containing 1% Triton X-100 (PBST). Tissues were then preadsorbed with 5% normal goat serum (Sigma) in PBST for 10 minutes and subsequently incubated with anti-DrmETH1 (1:500 dilution) for 2 days. After three washes with PBST (4 times for 15 minutes), tissues were incubated overnight in goat anti–rabbit IgG antibody conjugated with Alexa Fluor 488 (Molecular Probes). Images were captured in an epifluorescence microscope (Nikon E800).

## Author Contributions

H.J., Z.W. performed the experiments. R.J.N. provided the reagents. H.J., M.E.A., Y.P. analyzed the data. Y.P., H.J., R.J.N. and M.E.A. wrote the paper. Y.P. supervised the project.

## Supplementary Material

Supplementary Information Supplementary Dataset 1

## Figures and Tables

**Table 1:** Primers used in this study

Genes	GenBank Accession Number	Primers for cloning (5′ to 3′)	Primers for qPCR
TcPKr-A	KJ435303	F: ATGGACGATTACTACGTCAA	TGTCTGTAAAGTTTCGTGAGGC
	Tc011171	R: CTAGCTGTAGATCTCCCTCC	ATTCTAGGGATGTTTTTTGGGT
TcPKr-B	KJ435304	F: CATGCAAACAATGATCGTC	CATCTACGGCGAGCAAACT
	Tc011320	R: TTAATTGACCGAGACTGC	TCTGGCGGCTGTAAGAATC
TcPKr-C	KJ435305	F: ATGAGTCATCTCTGGAACGA	CGTCCATCCCAAACCTACTT
	Tc011318	R:CTA GGTGATATCTTCGTTGA	TCTCCTGTCTAAAAAACTTCACTG
TcCAPAr	KJ435306	F: ATGGACAACGATACAAAAGT	CGTCCACTATTTGTTTTACCCT
	Tc007170	R: TTATATTGGCGTTTCGTTGT	CCTCCTGCTGTCCCTATGC
TcETHr-B	EF222294	F: CTGACCCCCAATCCTACTATG	CTGCTTGTTAGCCTGGTTCA
	TC012494	R: CTCTAGACGAAAATCTCCCC	AAAAAGACGCAAGGGAGGG
